# A critical interpretive synthesis of the intersection of domestic violence with parental issues of mental health and substance misuse

**DOI:** 10.1111/hsc.12978

**Published:** 2020-03-23

**Authors:** Jasmin Isobe, Lucy Healey, Cathy Humphreys

**Affiliations:** ^1^ Department of Social Work Melbourne School of Health Sciences University of Melbourne Melbourne Vic. Australia

**Keywords:** child protection, children and families, critical interpretive synthesis, domestic and family violence, mental health, social work practice, substance misuse

## Abstract

A critical interpretive synthesis (CIS) methodology was used with the aim of informing practice with children and families when domestic and family violence (DFV) and parental issues relating to alcohol and other drugs (AOD) and mental health (MH) are also present. A CIS is grounded *in* the literature, but includes questioning *of* the literature in order to problematise gaps, contradictions and constructions of issues. A review of the literature from 2010 to 2018 was conducted with the structured search strategy identifying 40 relevant research articles. Synthesis and critique of these articles revealed three mutually informative themes through which to understand the literature and how it can inform practice. They were as follows: differences in theoretical approaches and client focus; complexity of system's collaboration; and practices converging on mothers. Taken together, these themes facilitated the development of the synthesising construct: *strengthening intersection between DFV, AOD and MH sectors*. Attention to practice at multiple levels that responds to the dynamics of gender and the differing impacts of violence was often lacking, particularly in the context of heightened child protection concerns where collaboration between sectors is needed. Both promising and problematic practices relating to gender dynamics and accountability converged on mothers. While there were exceptions, generally, there was an absence of engagement with, and recognition of, the impacts of fathers’ patterns of using violence and control on adult and child survivors. Promising practice related to the strengthening of the mother–child relationship and attention to MH and its intersection with domestic violence. Strengthening the intersections between DFV, AOD and MH practices with attention to keeping the perpetrator of violence in view is critical to overcoming the poor practice that can occur when sectors are siloed from each other.

**What is known about this topic**

- The intersection of domestic and family violence, mental health and substance misuse is complex and requires further exploration in terms of practice with children and families

**What this paper adds**

- A critical interpretive synthesis grounded in the international literature that engages with the complexity of practice at this intersection
- Exploration and critique of problematic and promising practices presented in the literature
- Synthesis of conceptual and practical issues towards strengthening practice with children and families at this intersection

## 1 INTRODUCTION

The co‐occurrence of domestic and family violence (DFV) with problems of mental health (MH) and alcohol and other drugs (AOD) is well established (Gilchrist, Hegarty, Chondros, Herman, & Gunn, 2010; Trevillion, Oram, Feder, & Howard, 2012). Each create difficulties for children living with one or both parents with these problems (Galvani, 2015; Kroll & Taylor, 2009). Where previous reviews have focused on these issues for women (Mason & O’Rinn, 2014), this article is focused on the nexus between DFV, MH and AOD when children are involved. Our interest has its roots in the practice issues for child protection and family service workers intervening with children and their families where there is DFV (Humphreys, Healey, & Mandel, 2018). While DFV is often the issue bringing children to the notice of child protection or family services, a case reading file analysis provides evidence of (usually) male‐perpetrated DFV sinking from view as the mother's MH or substance use become the focus of attention (Humphreys, Healey, Nicholson, & Kirkwood, 2018). This is not a new finding but suggests that practice in this area is entrenched.

A literature review was conducted to inform a research project between Australian researchers and the US‐based Safe & Together Institute. The *Safe & Together: Addressing ComplexitY,* (STACY) Project, undertook a review to inform DFV interventions where children are involved and where there are added issues of complexity, namely parental MH and AOD. To avoid conflating or replicating the notions of ‘multiproblem families’ or ‘troubled families’ where DFV, MH and AOD co‐occur, Dixon‐Woods et al.’s (2006) critical interpretive synthesis (CIS) was adopted as the most appropriate methodology for the review. The CIS enabled the authors to adopt a ‘DFV lens’ through which relevant literature was identified. In taking this approach, the authors sought evidence in the literature that services privileged the safety and well‐being of adult and child survivors by keeping the perpetrator (specifically, his pattern of behaviours and attitudes) in view, a perspective supported by the Safe & Together™ Model (Humphreys, Healey, Nicholson, et al., 2018). Further, a CIS allows literature to be problematised, thereby enabling critique that leads to synthesising arguments as opposed to descriptive or aggregative conclusions that characterise conventional systematic and scoping reviews (see Mason & O’Rinn, 2014). To this end, the following question was used to interrogate the literature:

*How does research into the intersection of domestic and family violence with mental health and alcohol and other drugs inform practice with children and families?*

## 2 METHODOLOGY

The CIS methodology, developed by Dixon‐Woods et al. (2006), has been employed to interrogate the literature on complex topics such as child sexual abuse (McKibbin, Humphreys, & Hamilton, 2016). It draws on conventions of qualitative research inquiry and systematic review methodology, enabling synthesis and critique of qualitative and quantitative evidence and discourse. In a CIS, a review question is formulated and guides rather than determines the review, acting as ‘a compass rather than an anchor’ (Dixon‐Woods et al., 2006, p. 37).

In addressing the guiding question, CIS draws on conventional systematic review techniques, such as the initial use of a structured search strategy. Selection criteria prioritise relevance to the research question and theory development, rather than the appraisal of evidence quality that underpins more traditional techniques of systematic review. A critical orientation recognises diverse ways of understanding the area under investigation. Some aspects of CIS may therefore not be reproducible, but the contribution provided by a critical orientation is complementary to conventional review methodology.

### 2.1 Paper selection and inclusion

A structured search strategy was used between June and August 2018 to access articles across five electronic databases: *CINAHL, Family & Society Studies Worldwide, MEDLINE, PsycINFO* and *SocINDEX.* Google Scholar was also accessed following the initial search.

Primary search term groups were refined in consultation with expert colleagues. Terms were grouped based on the main areas under investigation in the study (DFV, AOD and MH including mothers and fathers) with a further three term groups developed (dual diagnosis, social work practice and collaborative work). Table 1 provides an example of the search terms and combinations used to identify potentially relevant studies.

**TABLE 1:** Example search terms—Psycinfo record

1. (((domestic or family or interpersonal or intimate partner) adj (violen* or abus*)) or violence against women or gender‐based violence or (batter* adj wom#*n*)).ab,ti.
2. (alcohol* or drug* or addict* or "alcohol and other drugs" or AOD or SUD or (substance adj (abus* or addict* or use* or depend*))).ab,ti.
3. (mental health or mental illness or mental disorder* or mental health service* or MH or post‐traumatic stress or PTSD or mood disorder* or stress disorder* or depress* or anxiety).ab,ti.
4. ((dual diagnos* or comorbidity or co‐occur* or syndem* or (parental adj (mental ill‐health or mental health or issue* or violen* or substance abuse)) or mother* or women or father* or men) not HIV).ab,ti.
5. ((social adj (work* or practice* or service* or intervention* or support program)) or social work practice or best practice* or practitioner response* or practitioner perspective* or ((work* with or partner* with) adj2 (offending parent or non‐offending parent or mother* or women or father* or men or victim* or survivors* or perpetrator* offender* or abuser*))).ab,ti.
6. ((collaborat* or cooperat* or integrat* or network* or coordinat*) adj2 (work* or approach* or service* or practice* or intervention* or care or system* or initiative* or agency or multidiscipline*)).ab,ti.
7. 2 or 3
8. 1 and 7
9. 4 and 8
10. 5 or 6
11. 9 and 10

Table 2 shows the broad inclusion and exclusion criteria used in the screening and selection process. The search was limited to titles and abstracts, English language and publication between 2010 and 2018. Studies published before this date were not included given significant changes in social work theory and practice (Harms, Connolly, & Maidment, 2018), and the relatively widespread emergence and impact of methamphetamine (Galbraith, 2015). The bibliographies of key authors in the fields under investigation were also searched for relevant articles (some of them outside the search dates) based on expert colleague input and prominence in citation lists within the field. Relevance and theoretical contribution were prioritised over research design, methodology or evidence quality (Dixon‐Woods et al., 2006).

**TABLE 2:** Inclusion and exclusion criteria used in screening and selection of papers

Inclusion	Exclusion
Addressed the intersection of domestic and family violence with parental issues of alcohol and other drugs or mental health Addressed elements of practice at this intersection Addressed these issues in the context of working with children and families Context of research being relatable to Australian context Refereed journal articles	Did not address intersection of domestic and family violence with parental issues of alcohol and other drugs or mental health Did not focus on elements of practice at this intersection, that is had no focus on practice, or made only brief recommendations for practice Did not address the family context, that is focused only on women or men without children (explicitly or implicitly without mention of children as a factor for participants) Contextually disparate from Australian context Protocol papers, books, book reviews, newsletters, poster presentations, grey literature

Screening and sampling processes progressed concurrently in line with Dixon‐Woods et al.’s (2006) CIS methodology. This allowed for a rigorous search to be conducted that did not exclude potentially relevant research not accessible through a database search protocol alone. All search results were screened to determine potential inclusion in the synthesis. Screening involved one of the authors reading all abstracts of identified papers from all sources and creating lists of ‘potential’ articles for inclusion to be checked with the other authors. The full‐text PDFs of articles identified for potential inclusion were sourced and imported into NVivo 11. These were read and coded in line with the review question, and with continual consultation and discussion in the research team to inform decisions on inclusion. Any literature mentioned within the full text that seemed relevant to the review question was followed up with a reading of the title and abstract, in line with Dixon‐Woods et al.’s (2006) iterative process of selection and critique.

**FIGURE 1:**
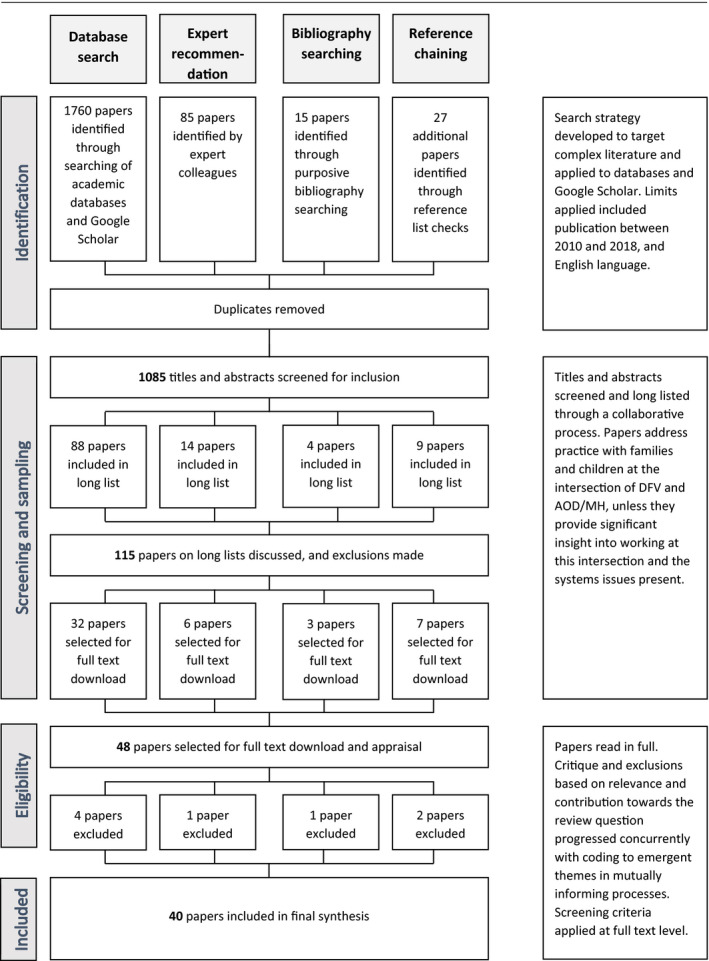
Paper selection process

Forty articles are included in the final synthesis (see Table 3), including qualitative and quantitative studies, systematic reviews and conceptual papers (See Figure 1). Throughout these processes, a log of procedures, in addition to reflective notes, was kept. These notes included emerging themes, gaps, contradictions and questions in relation to the literature.

**TABLE 3:** Papers included in final synthesis

Reference	Title of paper	Source	Methodology
Blythe et al. (2010)	Best Practices for Developing Child Protection Workers’ Skills: Domestic Violence, Substance Abuse, and Mental Health	Database search	Qualitative
Charles (2011)	Obstetricians and violence against women	Database search	Critical analysis
Choenni et al. (2017)	Association Between Substance Use and the Perpetration of Family Violence in Industrialised Countries: A Systematic Review	Expert recommendation	Systematic review
Coates (2017)	Working with families with parental mental health and/or drug and alcohol issues where there are child protection concerns: inter‐agency collaboration	Expert recommendation	Qualitative
Connelly et al. (2010)	A Model for Maternal Depression	Database search	Model description
Darlington et al. (2005)	Interagency collaboration between child protection and mental health services: Practices, attitudes and barriers.	Expert recommendation	Quantitative
Featherstone and Fraser (2012)	Working with Fathers around Domestic Violence: Contemporary Debates.	Reference chaining	Mixed methods
Frederico et al. (2014)	Child Protection and Cross‐Sector Practice: An Analysis of Child Death Reviews to Inform Practice When Multiple Parental Risk Factors Are Present	Database search	Mixed methods
Galvani (2015)	‘Drugs and relationships Don't Work’: Children's and Young People's Views of Substance Use and Intimate Relationships	Database search	Qualitative
Ghaffar et al. (2012)	Exploring the Experiences of Parents and Carers whose Children Have Been Subject to Child Protection Plans	Database search	Qualitative
Hashimoto et al. (2018)	Help‐seeking Behaviours for Intimate Partner Violence Perpetration by Men Receiving Substance Use Treatment: A mixed Methods Secondary Analysis	Database search	Mixed methods
Hegarty et al. (2013)	Screening and counselling in the primary care setting for women who have experienced intimate partner violence (WEAVE): a cluster randomised controlled trial	Database search	Quantitative
Holden et al. (2012)	Depressive Symptoms, Substance Abuse, and Intimate Partner Violence among Pregnant Women of Diverse Ethnicities	Database search	Quantitative
Holly and Horvath (2012)	A question of commitment – improving practitioner responses to domestic and sexual violence, problematic substance use and mental ill‐health	Bibliography search	Mixed methods
Howarth et al. (2016)	IMPRoving Outcomes for children exposed to domestic ViolencE (IMPROVE): an evidence synthesis	Database search	Mixed methods
Howell et al. (2015)	Strengthening Positive Parenting Through Intervention: Evaluating the Moms’ Empowerment Program for Women Experiencing Intimate Partner Violence	Database search	Quantitative
Humphreys and Thiara (2003)	Mental Health and Domestic Violence: ‘I Call it Symptoms of Abuse’	Expert recommendation	Qualitative
Lalayants (2013)	Multidisciplinary Collaboration on Child Protective Clinical Consultations: Perceptions of Best Practices	Database search	Qualitative
Laracuente (2017)	Therapeutic Engagement With Partner‐Abusive Fathers	Database search	Critical analysis
Loeffen et al. (2017)	Mentor mother support for mothers experiencing intimate partner violence in family practice: A qualitative study of three different perspectives on the facilitators and barriers of implementation	Database search	Qualitative
Macy and Goodbourn (2012)	Promoting Successful Collaborations Between Domestic Violence and Substance Abuse Treatment Service Sectors: A Review of the Literature	Expert recommendation	Systematic review
Macy et al. (2013)	Partner Violence and Substance Abuse Are Intertwined: Women's Perceptions of Violence‐Substance Connections	Reference chaining	Qualitative
Perera et al. (2014)	“It's Not That Straightforward”: When Family Support Is Challenging for Mothers Living With Mental Illness	Database search	Qualitative
Prosman et al. (2014)	Support by trained mentor mothers for abused women: a promising intervention in primary care	Database search	Quantitative
Radcliffe and Gilchrist (2016)	“You can never work with addiction in isolation”: Addressing intimate partner violence perpetration by men in substance misuse treatment	Database search	Qualitative
Rizo et al. (2018)	A Novel Intervention for System‐Involved Female Intimate Partner Violence Survivors: Changes in mental Health	Database search	Quasi‐experimental
Rose et al. (2011)	Barriers and facilitator of disclosures of domestic violence by mental health service users: qualitative study	Reference chaining	Qualitative
Sidebotham and Retzer (2018)	Maternal filicide in a cohort of English Serious Case Reviews	Database search	Mixed methods
Stover (2013)	Fathers for Change: A New Approach to Working With Fathers who Perpetrate Intimate Partner Violence	Database search	Intervention description
Stover et al. (2017)	Integrating intimate partner violence and parenting intervention into residential substance use disorder treatment for fathers	Database search	Mixed methods
Stover and Kiselica (2015)	Hostility and Substance Use in Relation to Intimate Partner Violence and Parenting Among Fathers	Database search	Quantitative
Stover et al. (2009)	Interventions for Intimate Partner Violence: Review and Implications for Evidence‐Based Practice	Bibliography search	Literature review
Taft et al. (2011)	Mothers’ AdvocateS In the Community (MOSAIC) – non‐professional mentor support to reduce intimate partner violence and depression in mothers: a cluster randomised trial in primary care	Database search	Quantitative
Templeton et al. (2009)	Young people living with parental alcohol misuse and parental violence: ‘No‐one has ever asked me how I feel in any of this’.	Reference chaining	Qualitative
Tsantefski et al. (2014)	Infant risk and safety in the context of maternal substance use	Database search	Qualitative
Tsantefski et al. (2015)	A delicate balance: intervention with mothers with dual diagnosis and their infants	Database search	Longitudinal mixed methods
Webber et al. (2013)	Inter‐agency joint protocols for safeguarding children in social care and adult mental‐health agencies: a cross‐sectional survey of practitioner experiences	Reference chaining	Mixed methods
Welland and Ribner (2010)	Culturally Specific Treatment for Partner‐Abusive Latino Men: A Qualitative Study to Identify and Implement Program Components	Database search	Qualitative
Willis et al. (2010)	Children Who Witness Violence: What Services Do They Need To Heal?	Database search	Qualitative
Zlotnick et al. (2011)	An interpersonally based intervention for low‐income pregnant women with intimate partner violence: a pilot study	Database search	Quantitative

### 2.2 Analysis and synthesis

Following Dixon‐Woods et al. (2006), analysis and synthesis of selected papers progressed concurrently. Analysis was undertaken in a similar way to that used in qualitative research. This involved iterative reading and coding of each text within NVivo 11, with nodes created inductively, and built on or collapsed as emergent themes were established. Content coded to nodes was read, during and following completion of full‐text reading, to inform the development of themes. Broad themes relating to the review question were generated following completion of first pass reading, considering ongoing critique and emerging subthemes within the literature. A synthesising construct was developed based on this analysis and critique.

### 2.3 Limitations

The nature of analysis and the ‘creative, interpretive processes involved’ (Dixon‐Woods et al., 2006, p. 40) does not lend itself to replicability. The collaborative processes used in selection and analysis of the literature instead required the authors to engage critically and reflexively with each other in the process of constructing a synthesised interpretation of the literature.

The grey literature, which describes practice initiatives, was not included and stands as a limitation of the review. Because articles that did not mention DFV are excluded, there is a lack of literature that concerns dual diagnosis and the learnings and insights from the extensive collaboration between the AOD and MH sectors (Glasby & Lester, 2004; Mastache, Mistral, Velleman, & Templeton, 2008).

## 3 FINDINGS

The CIS identified 40 diverse papers from the research literature that informed practice with families and children at the intersection of DFV and parental issues of AOD and/or MH. Our review was grounded *in* the literature but included questioning *of* the literature in order to problematise gaps, contradictions and constructions of issues towards informing practice for people working and living at the nexus of DFV and AOD and/or MH.

Initial reading and coding of emergent themes produced articles that included perspectives from clients, practitioners and researchers. Three mutually informative areas emerged through which to understand the literature and how it can inform practice. They are as follows: differences in theoretical approaches and client focus; complexity of system's collaboration; and practices converging on mothers. When these themes are taken together, they facilitated the development of our synthesising construct: *strengthening intersection between DFV, AOD and MH sectors*.

The findings are presented below through these three overarching themes with a final discussion about the prominence of topics in the literature. The synthesising construct is applied in the discussion to illustrate how the research can inform practice.

### 3.1 Differences in theoretical approach and focus

Engagement with the discourses across DFV, AOD and MH sectors revealed differences according to whether a gendered or de‐gendered theoretical approach informed client provision, and differences according to whether practice was adult‐ or child‐focused. Applying our synthesising construct, these two key areas of difference influenced the siloed way services interact on client issues of DFV, AOD and MH and have ramifications for practice at their intersection. Approaches to DFV, AOD and MH as separate issues have historically been adult‐focused, with children and child protection organisations only recently emerging as a priority within practice for these sectors (Holly & Horvath, 2012).

#### 3.1.1 Gendered, adult‐focused approaches

Approaches to DFV hold a gendered lens in order to identify who did what, to whom, and in what context, when engaging with clients and planning intervention and treatment. This relates to the well‐documented patterns of the gendered nature of DFV, in which men are the dominant perpetrators of violence against women, often with accruing impacts on the survivor's MH and substance use (Frederico, Jackson, & Dwyer, 2014). Applying the lens of the synthesising construct, acknowledgement of abusive and coercive behaviours as violence, and not just a relationship issue, distinguishes a DFV‐informed approach when there are co‐occurring problems with AOD and/or MH (Mandel, 2014). It highlights the need to shift problematic attitudes and beliefs when working with women experiencing DFV (Welland & Ribner, 2010). This is illustrated in the following quote in Humphreys and Thiara (2003):

‘I am irritated to this day that the people around me, that is, the health visitor, my social worker, his social worker, the GPs, in a way, all be it unwittingly, they perpetuated that myth in my head, because nobody else (until the domestic violence outreach worker) used the word ‘violence’.’ (participant, Humphreys & Thiara, 2003 p. 216)

Recognition of violent men as fathers is beginning to be addressed (Frederico et al., 2014); however, there is a distinct lack of focus on gender and fatherhood when it comes to programming for men with substance issues, with some exceptions (Stover, 2013; Stover, Carlson, & Patel, 2017).

#### 3.1.2 De‐gendered, adult‐focused approaches

The AOD and MH sectors are also adult‐focused but typically lack a gender lens. Mental health services attend to symptoms often through a diagnostic medical model, which lacks gendered nuance (Rose et al., 2011), and fails to understand that women's anxiety, depression, trauma reactions and suicide attempts may be ‘symptoms of abuse’ (Humphreys & Thiara, 2003). The AOD sector focuses on addiction and harm reduction (Tsantefski, Humphreys, & Jackson, 2014), often without considering possible factors relating to gender and DFV in the viability of treatment towards recovery (Macy, Renz, & Pelino, 2013). There are, however, promising signs of young people's views being considered and their voices brought to the conversation (Galvani, 2015; Templeton, Velleman, Hardy, & Boon, 2009).

There is also evidence of an emerging gendered lens in some AOD and MH services. In addition to the aforementioned newly emerging programs addressing DFV‐perpetrating fathers’ substance misuse, there are also programs for women as mothers with substance issues (Tsantefski, Jackson, & Humphreys, 2015), programs targeting maternal MH and amelioration of the mother–child bond in the context of DFV (Connelly, Baker‐Ericzen, Hazen, Landsverk, & Horwitz, 2010; Howell et al., 2015; Rizo, Wretman, Macy, Guo, & Ermentrout, 2018; Taft et al., 2011; Zlotnick, Capezza, & Parker, 2011). There was minimal exploration or differentiation of the different impacts and issues associated with alcohol and for individual drugs (Choenni, Hammink, & van de Mheen, 2017).

#### 3.1.3 De‐gendered, child‐focused approaches

Despite recognition of the complex intersection of DFV, AOD and MH, one‐dimensional approaches to working with families were consistently identified (Blythe, Heffernan, & Walters, 2010). Although DFV is often the catalyst for involvement with child protection services, effective service engagement for women is often at odds with the focus on the safety of the children (Sidebotham & Retzer, 2018; Tsantefski et al., 2015, p. 86). Paradoxically, this focus on risk and safety may mean that children's individual experiences and perspectives receive little to no attention (Templeton et al., 2009). As one young person put it, “no one has ever asked me about how I feel in any of this” (Templeton et al., 2009, p. 145). Thus, a heightened focus on children living with the intersecting complexities of DFV and parental AOD and MH emerges, while attention towards mothers’ needs and well‐being is diminished (Frederico et al., 2014; Radcliffe & Gilchrist, 2016; Tsantefski et al., 2015).

Active intervention with parents is further complicated by the fact that signs of risks to children may not be overt. In other words, assessments of the risks to children in the context of their parental and familial circumstances are missing. In analyses of child death reviews (Frederico et al., 2014) and child maltreatment fatalities (Sidebotham & Retzer, 2018), the authors highlighted the need to understand the history of DFV. Its invisibility meant that the mounting risks to children in the child protection system were not identified (Sidebotham & Retzer, 2018). Frederico et al. (2014) found that the majority of parents of children who died were found to have used ‘multiple substances’ yet,

‘…there appeared to be no systematic exploration of the effects of the combination of substances on parenting or its impact on the children.’ (Frederico et al., 2014, p. 109).

Findings also indicated the presence of a gender bias. In one study on child death reviews (Frederico et al., 2014), while workers had a stronger grasp of risks to children than to women, this did not translate into supporting mothers to address the impact of DFV, AOD or MH issues on individual children or the mother–child relationship. Instead, mothers were consistently the focus of engagement and monitoring, with concomitant disregard for father engagement and assessment of the impact of violence on family functioning and the child. The authors also note that ‘there was such an overwhelming lack of engagement with all of the men in the cases analysed that it was more suggestive of a gender bias’ than of worker fear of engagement (Frederico et al., 2014, p. 110). The escalating impact on the MH of women living with DFV was noted as critical for practitioners in responding to risks to children (Blythe et al., 2010; Perera, Short, & Fernabcher, 2014; Sidebotham & Retzer, 2018). Overall, there was reporting that the different sectors were siloed, with some exceptions when serious risks to children were identified.

A further example of gender bias and professional deflection is illustrated in the following quote in which responsibility for help‐seeking and initiation of support were often put back onto survivors of DFV:

‘Sometimes I ask myself, honestly, does it all belong to the family physician’s task, and do we have to do it all, inquire and feel inadequate if she does not disclose the abuse … they can ask me for anything, but patients have to take some initiative as well.’ (Family physician, female, age 59 in Loeffen et al., 2017, p. 30)

### 3.2 Complexity of system's collaboration

Not surprisingly, the “toxic trio” (Radcliffe & Gilchrist, 2016, p. 133) of DFV, AOD and MH as co‐occurring in families is a very strong theme in the literature (Frederico et al., 2014; Stover, Meadows, & Kaufman, 2009; Tsantefski et al., 2014). Heralding DFV as the “next frontier” (Holly & Horvath, 2012, p. 65) for MH and substance treatment services, much of the literature recognises the need for better integration across diverse programs and services (Stover et al., 2009) and the need for stronger collaborative relationships. Important areas of collaborative practice arising from the literature in this area have been underlined.

While most of the literature spoke of the benefits of collaborative working (Blythe et al., 2010; Lalayants, 2013), the challenges of siloed sectors were an equally strong theme from both client and practitioner perspectives (Coates, 2017; Frederico et al., 2014; Tsantefski et al., 2014; Webber, Mcree, & Angeli, 2013). There was recognition that no one strategy was effective; rather, in an area of complexity, multiple strategies were required (Macy & Goodbourn, 2012). This need for collaborative engagement was articulated by a survivor in Macy, Renz and Pelino's study (2013, p. 893):

'Yeah, [name of domestic violence program] were helpful, but they didn’t directly address the drug use. I guess that they relied on other agencies to take care of [survivor’s substance abuse problems]. But I think that had the shelter [staff] said to me, “Because of your history, it is required [for you to attend substance abuse treatment] and that you go to three NA [Narcotics Anonymous] meetings a week,” I think I would have had to do it. I think that could have benefited me. Also, it might have made a difference if we had had an NA meeting on site. Because I think that, absolutely the two [partner violence and substance abuse] are intertwined.’

Face‐to‐face meetings of professionals from different agencies and different disciplinary backgrounds were identified as good collaborative practice and actively supported. For example, multi‐agency training was seen to be beneficial, not only for the sharing of content areas, but also for the opportunity to meet and understand professionals with different knowledge bases and perspectives (Blythe et al., 2010). Networking events were cited as important (Holly & Horvath, 2012), as were multi‐agency meetings in which different professionals came together to negotiate collaborative arrangements across sectors (Radcliffe & Gilchrist, 2016; Sidebotham & Retzer, 2018).

Interagency training and events were noted as instrumental in overcoming barriers to partnership work, such as a lack of knowledge about partner agencies, stereotyping of other workers, unrealistic expectations of roles and poor communication between agencies (Coates, 2017; Darlington, Feeney, & Rixon, 2005). Articles reported on the workforce being under‐prepared for working across the different problem areas. Workers appeared to have greater confidence in working with women victims of domestic violence, but not necessarily with men, particularly if they were perpetrating violence (Radcliffe & Gilchrist, 2016). A point of contention was the lack of high‐quality, multi‐agency training and limited resources to facilitate these collaborative arrangements (Macy & Goodbourn, 2012).

At an operational level, the importance of co‐convened case planning meetings was highlighted pointing to the ability to tailor the response to meet the needs of individuals. However, it is notable that attention to fathers, even when still in the home, may be absent and points to a lack of collaboration between sectors (Tsantefski et al., 2014). Insufficient case conferences were identified in the analysis of child death reviews where issues of MH, AOD and DFV were nonetheless prominent (Frederico et al., 2014). Moreover, in those cases where the DFV was serious, the frequent absence of specialist women's DFV services at meetings was prominent.

Flowing on from the importance of opportunities for face‐to‐face engagement, several papers mentioned the significance of informal links between committed individuals who provided the lead or championing of collaborative partnerships (Holly & Horvath, 2012; Lalayants, 2013). In the early stages of adopting and integrating new practices, enthusiastic and influential individuals are particularly needed to provide leadership (Holly & Horvath, 2012). Team coordinators recognised this as an important part of their role; for example, (Lalayants, 2013, p.263):

‘I always use this analogy of the orchestra—we got drums there, we got wind instruments, and they all can play separately but then when they come together, they have to be able to harmonize, and if they can’t do it, they’re just going to make a lot of noise. It’s my job as a team coordinator to orchestrate them—to come in, step out, move up . . .’

While positive interpersonal relationships were seen as significant in overcoming barriers between services, role clarity of practitioners across different services was equally important in providing strong collaborative relationships (Coates, 2017; Darlington et al., 2005; Lalayants, 2013).

In addition to the importance of relationships between practitioners and agencies, protocols and formalised procedures (e.g. in relation to referrals and regularity of meetings) were recognised as critical (Coates, 2017; Lalayants, 2013; Webber et al., 2013). Protocols, for example, could be used as practice guides (Webber et al., 2013) in the sense that they define a set of steps to be taken to accomplish given tasks. Their formalisation provided confidence for the tasks involved in working across the different, interacting problems. That said, albeit with exceptions, they tended to focus on survivors, adult and child alike, rather than the work with perpetrators of violence. One notable exception is a model for safely engaging partner‐abusive fathers in their children's treatment, presented through a case vignette (Laracuente, 2017). The author offers a framework for engagement that focuses on safety first; getting the survivor's perspective; exploring the children's experiences; consultation with collaterals such as CP workers and lawyers involved in the case; getting to know the father's perspective; choosing a safe approach with strict parameters and achievable goals; and utilising a running list to document interaction with fathers (Laracuente, 2017).

There was a tension between the stated desire for formalisation of processes (including relationships), on the one hand, and recognition that strong personal relationships were required to overcome siloed working, and establishing and maintaining collaboration, on the other (Lalayants, 2013; Webber et al., 2013). The importance of senior management involvement with the necessary authority to make decisions to change practice or establish partnerships was also seen as salient (Darlington et al., 2005; Lalayants, 2013). Managers who would influence political leaders and policy makers to sustain collaborations in the long term were key to successful partnership working across DFV, AOD and MH services. Without senior management authorisation, partnerships could not sustain the inevitable loss of key actors (Holly & Horvath, 2012).

### 3.3 Practices converging on mothers

Practices converging on mothers are manifested on multiple levels, from theoretical and systems issues through to individual worker practices (Radcliffe & Gilchrist, 2016). A substantial number of articles concerned practice and interventions for mothers related to DFV and MH. In most cases, this research focused on women's MH linked to outcomes for their children (Connelly et al., 2010; Hegarty et al., 2013; Holden, McKenzie, Pruitt, Aaron, & Hall, 2012; Howarth et al., 2016; Howell et al., 2015; Loeffen et al., 2017; Perera et al., 2014; Prosman, Lo Fo Wong, & Lagro‐Janssen, 2014; Rizo et al., 2018; Taft et al., 2011; Zlotnick et al., 2011). In contrast, articles focused on interventions and practice with fathers featured less prominently within our sample, and for the most part concerned DFV and AOD (Hashimoto, Radcliffe, & Gilchrist, 2018; Laracuente, 2017; Radcliffe & Gilchrist, 2016; Stover, 2013; Stover, Carlson & Patel; Stover & Kiselica, 2015; Welland & Ribner, 2010). Featherstone and Fraser (2012) explore contemporary debates about working with domestically violent fathers. They note tensions in different theoretical approaches, such as those that view men as perpetrators and those that construct them as fathers, and point to practice level issues around delivery of set programs versus individualised responses. They also emphasise the need for further exploration and engagement with the additional issues of AOD and MH.

The relative lack of worker engagement with fathers is noted and discussed in terms of gender bias by Frederico et al. (2014). They identify increased compliance and assessment of “protectiveness” and monitoring of mothers, with little engagement towards her individual wellbeing (2014, p. 110). Practitioners in another study directly mention a gender gap in questioning around DFV in AOD contexts, exemplified below.

‘I think some more specialist training. And a chance to think about how we identify better but I think we’ve only got as far really as identifying in perpetrators, I think in victims we’re probably slightly better’ (Substance Misuse Practitioner quoted in Radcliffe & Gilchrist, 2016, p. 136)

Laracuente (2017, p. 384) provides a stark assessment:

‘This maternal focus in IPV intervention, although useful and necessary, reinforces victim blaming and leaves partner‐abusive fathers free from taking responsibility.’

One study recognised the actions and impacts of the abusive partner as a father in a residential substance misuse program (Stover et al., 2017), while another recognised that directly inquiring about relationship problems, to provide a space for men to disclose, could provide a practice opportunity (Hashimoto et al., 2018). Except for one study, there was little to show in terms of practice other than in relation to the development of a culturally specific program in southern California for Latino men who use violence and control (Welland & Ribner, 2010). The program addressed men's childhood trauma, alcohol abuse and parenting skills, while holding them to account for their use of violence and control towards their partners and children. The program was particularly responsive to challenging the men's stereotypical views of women as the sole nurturing parent in a family.

The gendered construction of parenthood points to asymmetrical expectations and standards and is illustrated through references to the idea of ‘bad mother’. This appears in the context of ideals of motherhood and gender norms (Charles, 2011) and capacity to parent children effectively in the context of AOD and MH issues (Perera et al., 2014; Tsantefski et al., 2015). Under the intense gaze of child protective services, fear related to disclosure of DFV or AOD issues emerged as a concern for parents, including those perpetrating abuse (Hashimoto et al., 2018). It was also a consistent theme across non‐offending parent accounts (Loeffen et al., 2017; Macy et al., 2013). Mothers expressed fear of being disbelieved, and of increased violence (Rose et al., 2011), but above all fear of child removal (Ghaffar, Manby, & Race, 2012; Macy et al., 2013; Tsantefski et al., 2014), as exemplified in women's experiences with practitioners from, respectively, statutory child protection and specialist alcohol and other drug obstetric service:

‘Maybe had I known that [even if I were] admitting drug use, they’re not going to take my kids away, or I’m admitting being in an abusive [partner violence] situation, they’re not going to take my kids away either. Had they just said, “We’re going to get you the help you need, and we’re not going to take your kids,” that would have made all the difference in the world to me.’ (Macy et al., 2013, pp. 892–893)

‘I'd love to say to somebody, “I need help in this or that regard”, but they're going to think that I'm not coping and that I can't look after her.’ (Tsantefski et al., 2014, p. 13)

These fears acted as significant barriers to help‐seeking, even if they were based in mothers’ best efforts to keep their children safe (Tsantefski et al., 2015), and highlighted the complexity and dynamics behind disclosure and non‐disclosure. While women were concerned about child protection involvement, interesting examples emerged of successful, non‐professional mentoring of women with babies and young children struggling with MH and DFV issues. Loeffen et al. (2017) report on a promising mentor mother support intervention in which ‘paraprofessional friends’ mentor survivor mothers. There are similarities with the MOSAIC project which also focused on a non‐professional mentor to support women to reduce both DFV and depression (Taft et al., 2011). These were in line with calls from women for better services for them as mothers that would then in turn help to address the impact of the violence on their children. One mother articulates the connection as the “father bashes the mother but it hurts the kids!” (Willis et al., 2010, p. 556). These women, along with children, also emphasised the importance of programs and services specifically for children and young people that are based on respect and support (Willis et al., 2010), that uphold the resilience of children and respond to their needs as individuals (Templeton et al., 2009).

### 3.4 Topic prominence

As the development of our themes and findings presented above progressed, a brief examination of the prominence of topic words was conducted as a complementary exercise. Using the word frequency function in NVivo, we determined the most frequent words present in the content coded as relevant to our review. The 10 most frequently used words were: (a) violence, (b) women, (c) children, (d) substance, (e) abuse, (f) IPV, (g) health, (h) services, (i) mental, (j) family. Expanding this to the 150 most frequently used words, we used the word cloud visualisation function to generate the word cloud below—higher frequency words appear larger, with less frequently used words appearing as smaller. We found this to be quite compelling, and reflective of the themes and concepts we had developed through analysis.

**FIGURE 2:**
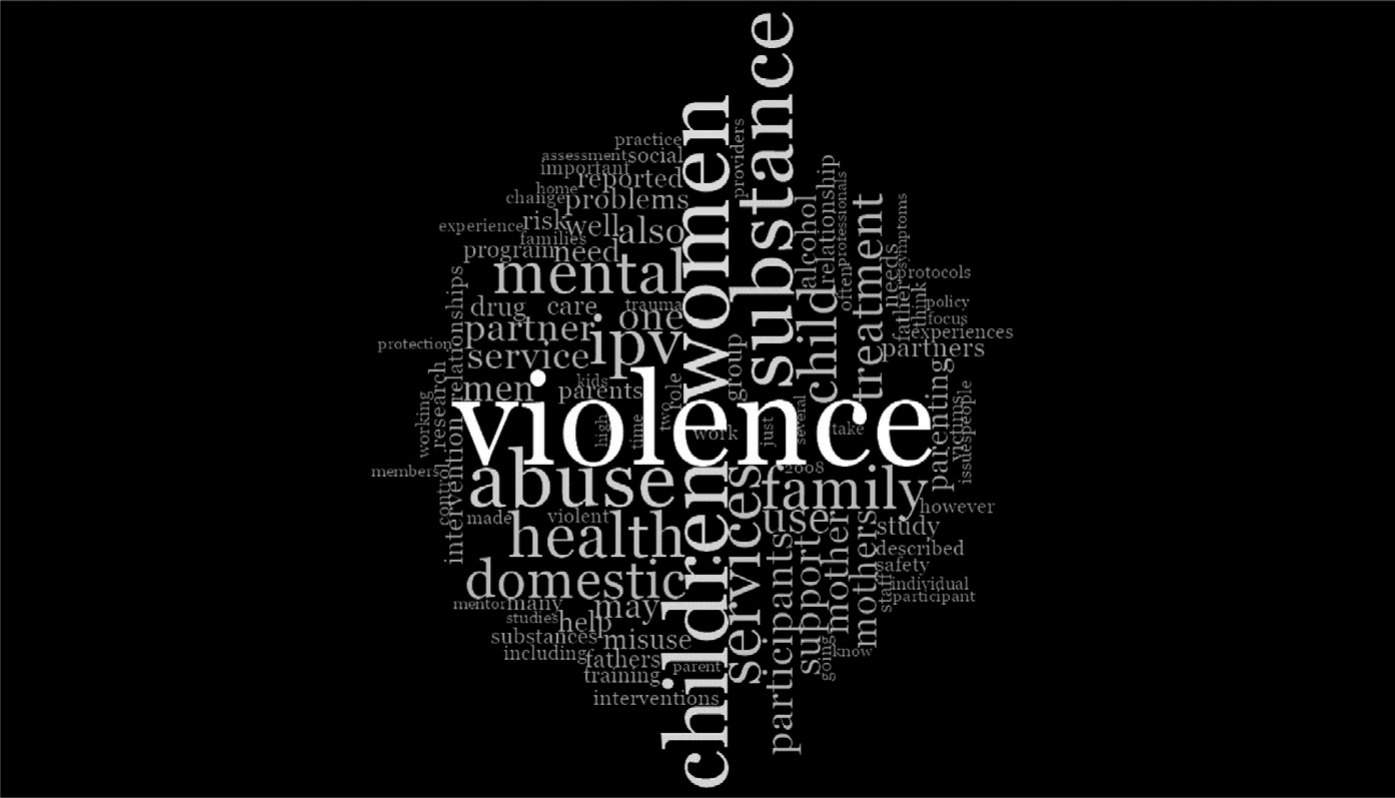
Word cloud of article content

The authors noted in particular: the prominence of the issue of violence alongside women and children, standing out starkly, but surrounded by grey areas and a myriad of factors for consideration; prominence of ‘women’ in relation to ‘mother,’ and the contrasting size of ‘mother’ relative to ‘father’, ‘men’ or ‘parenting’; and words such as ‘co‐occurrence’ and ‘integration’ cannot be found (see Figure 2).

## 4 DISCUSSION

The synthesising construct—*strengthening intersection between DFV, AOD and MH sectors*—was derived from the literature. The CIS particularly explored the ways in which the gendered dynamics of DFV informed AOD and MH practices. These included: keeping the domestic violence perpetrator in view; supporting the safety and well‐being of survivors including their strategies of resistance to violence and abuse; and recognising the harm to children flowing from the perpetrator's tactics of abuse, including the undermining of the child's relationship with their mother (Humphreys, Healey, & Mandel, 2018).

The graphic drawn from NVivo of the word frequencies when searching the relevant literature highlights the focus of practice and research when there are co‐occurring parental problems of domestic violence, substance misuse and/or mental health. Writ large, as would be expected, is ‘violence’. The prominence of ‘women’ and ‘children’ is striking, in comparison to the diminished scale of ‘men’ and ‘fathers’. The words ‘perpetrator’ and ‘offender’ are so infrequent as to not feature. This exercise does not give the context these terms are used in, providing only a high‐level indication of the focus of these articles. However, in light of the analysis undertaken and the findings presented in the previous section, it does provide a striking visual representation of the emergent themes.

An important issue of intervention where there is DFV is to pivot the practice to ensure that the perpetrator is kept in view (Mandel, 2014), that the behaviours and patterns of coercive control are explored and the impact of this violence on the non‐offending parent and children is understood. In the first instance, this means that DFV needs to be identified and appropriate responses provided. In the literature, there was recognition of the need to expand inquiry to all family members in order to identify appropriate intervention. However, it was clear that there was reluctance from many professionals, particularly in the AOD and MH areas, to make the most basic enquiries about the man's relationship to his family members (Radcliffe & Gilchrist, 2016). Some professionals recognised that organisations supporting people with substance misuse were well placed to respond to fathers who use violence given the co‐occurrence of their substance use with their use of violence (Hashimoto et al., 2018), and the associated increase in severity of violence when they are using drugs or alcohol (Humphreys, Regan, Rivers, & Thiara, 2005).

There was a distinct absence in the articles under review of the way in which a man's MH issues interacted with his use of violence. It is an issue raised only in relation to women as mothers (Perera et al., 2014). The perpetrator's MH issues (including threatening suicide) are part of many standardised DFV risk assessment tools (e.g. SARA, DVRNA, Danger Assessment) given the association with lethality. However, there does not appear to be a concerted effort to address this issue in MH services. The issues for children when their fathers are both violent and struggling with MH issues are rarely mentioned in the literature under review in this CIS.

There is, however, a significant focus on the mother's MH and its impact on her ability to look after her children, with children's wellbeing often linked to their mother's when there are issues of DFV (Connelly et al., 2010; Holden et al., 2012; Howarth et al., 2016; Loeffen et al., 2017; Perera et al., 2014; Prosman et al., 2014; Zlotnick et al., 2011). As Sullivan (2007) points out, the intervening variable may be the violent man that adult and child survivors are both living with and whose pattern of behaviour is creating fear and trauma. It is an area where the invisibility of perpetrator behaviour is particularly marked.

The focus on women's MH reified from the violence that they have experienced is a particularly strong pattern, though one which is now being consistently identified in the literature as problematic (Humphreys & Thiara, 2003; Sidebotham & Retzer, 2018). Nevertheless, until MH services and child protection organisations shift their focus to the perpetrator of violence as the source of many of the disturbing problems in children and their mothers, it will be difficult to make meaningful practice changes. Some ideas for re‐focusing the work are provided through the Safe & Together™ Model (Mandel, 2014) or the Practice‐Based Response, which focuses on the ways in which women resist the violence to which they and their children are subjected (Wade, 1997).

The intersection between DFV, AOD and MH can be pernicious for women. While there are some programs that have developed supportive responses to women living with co‐occurring problems (Taft et al., 2011; Tsantefski et al., 2015), the reports from women are that they have an immense fear of the removal of their children should they disclose the complexity of problems they are experiencing (Macy et al., 2013). Child protection workers are often perceived by women to be monitoring the woman's MH, use of substances, and ability to protect their children from the perpetrator of violence (Frederico et al., 2014; Tsantefski et al., 2014), rather than providing support and actively intervening with the perpetrator of violence. Some professionals blame women for not proactively seeking help and perceived them as difficult and uncooperative (Loeffen et al., 2017). Under these circumstances, the isolating tactics associated with domestic violence are compounded at the level of the service system and that of women's and children's informal networks. It is where questions need to be raised about the safety of the service system response and whether it is replicating abusive tactics or providing an appropriate response to safety and wellbeing for women and children survivors (Heward‐Belle, Humphreys, Laing, & Toivonen, 2018).

Children have a range of ways of indicating their distress at different ages and developmental stages. The ways children respond to living with DFV (Kimball, 2016; McTavish, MacGregor, Wathen, & MacMillan, 2016) show similar symptoms to children living with substance use (Kroll & Taylor, 2009). It is here that the number of Adverse Childhood Experiences (ACEs) that children experience is relevant (Oral et al., 2016), particularly when it is recognised that living with DFV is the strongest predictor of other adverse experiences (McGavock & Spratt, 2017). While this review did not focus on ACEs and practices to address these specifically, the issues which confront children highlight the need for a more proficient and nuanced response to intervening where there are complex, co‐occurring problems with their mothers and/or fathers. This includes increasing attention and genuine engagement with the voices of children who are living with DFV and parental issues of AOD and MH. This emerged as a limitation and an area for a future review. As presented in the previous section, there is an increased focus on risk and safety as it applies to children living with these complexities that manifest in service responses to their parents (particularly and often problematically for mothers).

While the nature of the CIS analysis is not directly replicable, noted as a potential limitation in previous sections, this methodology has facilitated critical engagement and attention to aspects of discourse across the literature that would not be possible through more conventional aggregative methodologies. The areas of practice, highlighted and problematised that contribute to the synthesising construct of this review, *strengthening intersection between DFV, AOD and MH sectors*, have significant potential to inform practice with families living at this complex intersection.

## 5 CONCLUSION

The review of the literature points to areas in which there are some promising practices emerging in response to the co‐occurrence of DFV, AOD and MH (Holly & Horvath, 2012; Laracuente, 2017; Stover et al., 2009; Taft et al., 2011). It is also clear that the service system response is at a relatively early stage in managing complexity, especially given the absence of a gendered, DFV‐informed, child‐focussed approach to understanding the risks to children in the context of parental AOD and/or MH problems. The impact of DFV too easily disappears when other problems emerge, particularly when these involve the child's mother. The absent presence of the perpetrator of violence (Thiara & Humphreys, 2017) needs to be addressed wherever he appears within the service system. Until practices are developed in MH and AOD services to identify and respond to DFV—specifically fathers who use violence—and the intersection between DFV, AOD and MH sectors is strengthened, the lives of women and children may not improve.

## References

[hsc12978-bib-0001] Blythe, B. , Heffernan, K. , & Walters, B. (2010). Best practices for developing child protection workers' skills: Domestic violence, substance abuse, and mental health training. Social Work Review/Revista De Asistenta Sociala, 9(2), 51–64.

[hsc12978-bib-0002] Charles, S. (2011). Obstetricians and violence against women. The American Journal of Bioethics: AJOB, 11(12), 51–56. 10.1080/15265161.2011.623813 22146035

[hsc12978-bib-0003] Choenni, V. , Hammink, A. , & van de Mheen, D. (2017). Association between substance use and the perpetration of family violence in industrialized countries: A systematic review. Trauma, Violence, & Abuse, 18(1), 37–50. 10.1177/1524838015589253 26296740

[hsc12978-bib-0004] Coates, D. (2017). Working with families with parental mental health and/or drug and alcohol issues where there are child protection concerns: Inter‐agency collaboration. Child & Family Social Work, 22, 1–10.

[hsc12978-bib-0005] Connelly, C. D. , Baker‐Ericzen, M. J. , Hazen, A. L. , Landsverk, J. , & Horwitz, S. M. (2010). A model for maternal depression. Journal of Women's Health, 19(9), 1747–1757. 10.1089/jwh.2009.1823 PMC2965697 20718624

[hsc12978-bib-0006] Darlington, Y. , Feeney, J. , & Rixon, K. (2005). Interagency collaboration between child protection and mental health services: Practices, attitudes and barriers. Child Abuse & Neglect, 29(10), 1085–1098. 16315352 10.1016/j.chiabu.2005.04.005

[hsc12978-bib-0007] Dixon‐Woods, M. , Cavers, D. , Agarwal, S. , Annandale, E. , Arthur, A. , Harvey, J. , … Sutton, A. J. (2006). Conducting a critical interpretive synthesis of the literature on access to healthcare by vulnerable groups. BMC Medical Research Methodology, 6, 35 10.1186/1471-2288-6-35 16872487 PMC1559637

[hsc12978-bib-0008] Featherstone, B. , & Fraser, C. (2012). Working with fathers around domestic violence: Contemporary debates. Child Abuse Review, 21, 255–263.

[hsc12978-bib-0009] Frederico, M. , Jackson, A. , & Dwyer, J. (2014). Child protection and cross‐sector practice: An analysis of child death reviews to inform practice when multiple parental risk factors are present. Child Abuse Review, 23(2), 104–115. 10.1002/car.2321

[hsc12978-bib-0010] Galbraith, N. (2015). The methamphetamine problem: Commentary on … Psychiatric morbidity and socio‐occupational dysfunction in residents of a drug rehabilitation centre. British Journal of Psychiatry Bulletin, 39(5), 218–220. 10.1192/pb.bp.115.050930 PMC4706185 26755964

[hsc12978-bib-0011] Galvani, S. (2015). 'Drugs and relationships don't work': Children's and young people's views of substance use and intimate relationships. Child Abuse Review, 24(6), 440–451. 10.1002/car.2292

[hsc12978-bib-0012] Ghaffar, W. , Manby, M. , & Race, T. (2012). Exploring the experiences of parents and carers whose children have been subject to child protection plans. British Journal of Social Work, 42(5), 887–905. 10.1093/bjsw/bcr132

[hsc12978-bib-0013] Gilchrist, G. , Hegarty, K. , Chondros, P. , Herman, H. , & Gunn, J. (2010). The association between intimate partner violence, alcohol and depression in family practice. BMC Family Practice, 11, 72 10.1186/1471-2296-11-72 20868526 PMC2954955

[hsc12978-bib-0014] Glasby, J. , & Lester, H. (2004). Cases for change in mental health: Partnership working in mental health services. Journal of Interprofessional Care, 18(1), 7–16. 10.1080/13561820410001639316 14668098

[hsc12978-bib-0015] Harms, L. , Connolly, M. , & Maidment, J. (2018). Social Work: Contexts and Practice (4th ed.). Oxford: Oxford University Press.

[hsc12978-bib-0016] Hashimoto, N. , Radcliffe, P. , & Gilchrist, G. (2018). Help‐seeking behaviors for intimate partner violence perpetration by men receiving substance use treatment: A mixed‐methods secondary analysis. Journal of Interpersonal Violence, 886260518770645 10.1177/0886260518770645 29756559

[hsc12978-bib-0017] Hegarty, K. , O'Doherty, L. , Taft, A. , Chondros, P. , Brown, S. , Valpied, J. , … Gunn, J. (2013). Screening and counselling in the primary care setting for women who have experienced intimate partner violence (WEAVE): A cluster randomised controlled trial. Lancet (London, England), 382(9888), 249–258. 10.1016/S0140-6736(13)60052-5 23598181

[hsc12978-bib-0018] Heward‐Belle, S. , Humphreys, C. , Laing, L. , & Toivonen, C. (2018). Intervening with children living with domestic violence: Is the system safe? Australian Social Work, 71, 135–147.

[hsc12978-bib-0019] Holden, K. B. , McKenzie, R. , Pruitt, V. , Aaron, K. , & Hall, S. (2012). Depressive symptoms, substance abuse, and intimate partner violence among pregnant women of diverse ethnicities. Journal of Health Care for the Poor and Underserved, 23(1), 226–241. 10.1353/hpu.2012.0022 22643473 PMC3401533

[hsc12978-bib-0020] Holly, J. , & Horvath, M. A. H. (2012). A question of commitment – improving practitioner responses to domestic and sexual violence, problematic substance use and mental ill‐health. Advances in Dual Diagnosis, 5(2), 59–67. 10.1108/17570971211241912

[hsc12978-bib-0021] Howarth, E. , Moore, T. H. M. , Welton, N. J. , Lewis, N. , Stanley, N. , MacMillan, H. , … Feder, G. (2016). IMPRoving Outcomes for children exposed to domestic ViolencE (IMPROVE): an evidence synthesis. Public Health Research, 4(10), 1–342. 10.3310/phr04100 27977089

[hsc12978-bib-0022] Howell, K. H. , Miller, L. E. , Lilly, M. M. , Burlaka, V. , Grogan‐Kaylor, A. C. , & Graham‐Bermann, S. A. (2015). Strengthening positive parenting through intervention: Evaluating the Moms' Empowerment Program for women experiencing intimate partner violence. Journal of Interpersonal Violence, 30(2), 232–252. 10.1177/0886260514533155 24832954

[hsc12978-bib-0023] Humphreys, C. , Healey, L. , & Mandel, D. (2018). Case reading as a practice and training intervention in domestic violence and child protection. Australian Social Work, 71, 162–174. 10.1080/0312407X.2017.1413666

[hsc12978-bib-0024] Humphreys, C. , Healey, L. , Nicholson, D. , & Kirkwood, D. (2018). Making the case for a differential child protection response for children living with domestic and family violence. Australian Social Work, 71, 162–174. 10.1080/0312407X.2017.1415366

[hsc12978-bib-0025] Humphreys, C. , Regan, L. , Rivers, D. , & Thiara, R. K. (2005). Domestic violence and substance misuse: Tackling complexity. British Journal of Social Work, 35(7), 1303–1320.

[hsc12978-bib-0026] Humphreys, C. , & Thiara, R. K. (2003). Mental health and domestic violence: 'I call it symptoms of abuse'. British Journal of Social Work, 33(2), 209–226.

[hsc12978-bib-0027] Kimball, E. (2016). Edleson revisited: Reviewing children’s witnessing of domestic violence 15 years later. Journal of Family Violence, 31, 625–637.

[hsc12978-bib-0028] Kroll, B. , & Taylor, A. (2009). Interventions for children and families where there is parental drug misuse. London, UK: London School of Hygiene and Tropical Medicine.

[hsc12978-bib-0029] Lalayants, M. (2013). Multidisciplinary collaboration in child protective clinical consultations: Perceptions of best practices. Journal of Public Child Welfare, 7(3), 253–274. 10.1080/15548732.2013.798245

[hsc12978-bib-0030] Laracuente, M. S. (2017). Therapeutic engagement with partner‐abusive fathers. Family Journal, 25(4), 383–388. 10.1177/1066480717731221

[hsc12978-bib-0031] Loeffen, M. J. W. , Daemen, J. , Wester, F. P. J. F. , Laurant, M. G. H. , Lo Fo Wong, S. H. , & Lagro‐Janssen, A. L. M. (2017). Mentor mother support for mothers experiencing intimate partner violence in family practice: A qualitative study of three different perspectives on the facilitators and barriers of implementation. The European Journal of General Practice, 23(1), 27–34. 10.1080/13814788.2016.1267724 28095727 PMC5774271

[hsc12978-bib-0032] Macy, R. J. , & Goodbourn, M. (2012). Promoting successful collaborations between domestic violence and substance abuse treatment service sectors: A review of the literature. Trauma, Violence, & Abuse, 13(4), 234–251. 10.1177/1524838012455874 22899704

[hsc12978-bib-0033] Macy, R. J. , Renz, C. , & Pelino, E. (2013). Partner violence and substance abuse are intertwined: Women's perceptions of violence‐substance connections. Violence against Women, 19(7), 881–902. 10.1177/1077801213498208 23955931

[hsc12978-bib-0034] Mandel, D. (2014). Beyond domestic violence perpetrator accountability in child welfare systems. The no to Violence Journal, Spring, 50–85.

[hsc12978-bib-0035] Mason, R. , & O’Rinn, S. E. (2014). Co‐occurring intimate partner violence, mental health, and substance use problems: A scoping review. Global Health Action, 7, 1–17. 10.3402/gha.v7.24815 PMC4240863 25416321

[hsc12978-bib-0036] Mastache, C. , Mistral, W. , Velleman, R. , & Templeton, L. (2008). Partnership working in community alcohol prevention programmes. Drugs: Education, Prevention & Policy, 15, 4–14.

[hsc12978-bib-0037] McGavock, L. , & Spratt, T. (2017). Children exposed to domestic violence: Using adverse childhood experience scores to inform service response. British Journal of Social Work, 47, 1128–1146.

[hsc12978-bib-0038] McKibbin, G. , Humphreys, C. , & Hamilton, B. (2016). Prevention‐enhancing interactions: A Critical Interpretive Synthesis of the evidence about children who sexually abuse other children. Health & Social Care in the Community, 24(6), 657–671. 10.1111/hsc.12260 26094766

[hsc12978-bib-0039] McTavish, J. R. , MacGregor, J. C. D. , Wathen, N. , & MacMillan, L. (2016). Children’s exposure to intimate partner violence: An overview. International Review of Psychiatry, 28(5), 504–518. 27414209 10.1080/09540261.2016.1205001

[hsc12978-bib-0040] Oral, R. , Ramirez, M. , Coohey, C. , Nakada, S. , Walz, A. , Kuntz, A. , … Peek‐Asa, C. (2016). Adverse childhood experiences and trauma informed care: The future of health care. Pediatric Research, 79, 227–233. 26460523 10.1038/pr.2015.197

[hsc12978-bib-0041] Perera, D. N. , Short, L. , & Fernbacher, S. (2014). "It's not that straightforward": When family support is challenging for mothers living with mental illness. Psychiatric Rehabilitation Journal, 37(3), 170–175. 10.1037/prj0000074 24866838

[hsc12978-bib-0042] Prosman, G.‐J. , Lo Fo Wong, S. H. , & Lagro‐Janssen, A. L. M. (2014). Support by trained mentor mothers for abused women: A promising intervention in primary care. Family Practice, 31(1), 71–80. 10.1093/fampra/cmt058 24132592

[hsc12978-bib-0043] Radcliffe, P. , & Gilchrist, G. (2016). "You can never work with addictions in isolation": Addressing intimate partner violence perpetration by men in substance misuse treatment. The International Journal on Drug Policy, 36, 130–140. 10.1016/j.drugpo.2016.03.010 27107548

[hsc12978-bib-0044] Rizo, C. F. , Wretman, C. J. , Macy, R. J. , Guo, S. , & Ermentrout, D. M. (2018). A novel intervention for system‐involved female intimate partner violence survivors: Changes in mental health. American Journal of Orthopsychiatry, 88(6), 681–690. 10.1037/ort0000332 30024179

[hsc12978-bib-0045] Rose, D. , Trevillion, K. , Woodall, A. , Morgan, C. , Feder, G. , & Howard, L. (2011). Barriers and facilitators of disclosures of domestic violence by mental health service users: Qualitative study. The British Journal of Psychiatry, 198, 189–194. 21160053 10.1192/bjp.bp.109.072389

[hsc12978-bib-0046] Sidebotham, P. , & Retzer, A. (2018). Maternal filicide in a cohort of English serious case reviews. Archives of Women's Mental Health, 10.1007/s00737-018-0820-7 PMC6373272 29500658

[hsc12978-bib-0047] Stover, C. S. (2013). Fathers for change: A new approach to working with fathers who perpetrate intimate partner violence. Journal of the American Academy of Psychiatry and the Law, 41(1), 65–71. 23503178 PMC3641144

[hsc12978-bib-0048] Stover, C. S. , Carlson, M. , & Patel, S. (2017). Integrating intimate partner violence and parenting intervention into residential substance use disorder treatment for fathers. Journal of Substance Abuse Treatment, 81, 35–43. 10.1016/j.jsat.2017.07.013 28847453

[hsc12978-bib-0049] Stover, C. S. , & Kiselica, A. (2015). Hostility and substance use in relation to intimate partner violence and parenting among fathers. Aggressive Behavior, 41(3), 205–213. 10.1002/ab.21548 25043704 PMC4710568

[hsc12978-bib-0050] Stover, C. S. , Meadows, A. L. , & Kaufman, J. (2009). Interventions for intimate partner violence: Review and implications for evidence‐based practice. Professional Psychology: Research and Practice, 40(3), 223–233. 10.1037/a0012718

[hsc12978-bib-0051] Sullivan, C. (2007). Evaluating parenting programs for men who batter: Current considerations and controversies In Eldleson J., & Williams O. (Eds.), Parenting by Men Who Batter (pp. 137–148). Oxford: Oxford University Press.

[hsc12978-bib-0052] Taft, A. J. , Small, R. , Hegarty, K. L. , Watson, L. F. , Gold, L. , & Lumley, J. A. (2011). Mothers' AdvocateS In the Community (MOSAIC)‐ non‐professional mentor support to reduce intimate partner violence and depression in mothers: A cluster randomised trial in primary care. BMC Public Health, 11(1), 178–187. 10.1186/1471-2458-11-178 21429226 PMC3074545

[hsc12978-bib-0053] Templeton, L. , Velleman, R. , Hardy, E. , & Boon, S. (2009). Young people living with parental alcohol misuse and parental violence: ‘No‐one has ever asked me how I feel in any of this’. Journal of Substance Use, 14(3–4), 139–150.

[hsc12978-bib-0054] Thiara, R. K. , & Humphreys, C. (2017). Absent presence: The on‐going impact of men’s violence on the mother‐child relationship. Child and Family Social Work Social Work, 22, 137–145. 10.1111/cfs.12210

[hsc12978-bib-0055] Trevillion, K. , Oram, S. , Feder, G. , & Howard, L. M. (2012). Experiences of domestic violence and mental disorders: A systematic review and meta‐analysis. PLoS ONE, 7(12), e51740 10.1371/journal.pone.0051740 23300562 PMC3530507

[hsc12978-bib-0056] Tsantefski, M. , Humphreys, C. , & Jackson, A. C. (2014). Infant risk and safety in the context of maternal substance use. Children & Youth Services Review, 47, 10–17. 10.1016/j.childyouth.2013.10.021

[hsc12978-bib-0057] Tsantefski, M. , Jackson, A. C. , & Humphreys, C. (2015). A delicate balance: Intervention with mothers with dual diagnosis and their infants. Advances in Dual Diagnosis, 8(2), 78–89. 10.1108/ADD-09-2014-0027

[hsc12978-bib-0058] Wade, A. (1997). Small acts of living: Everyday resistance to violence and other forms of oppression. Contemporary Family Therapy, 19(1), 23–39.

[hsc12978-bib-0059] Webber, M. , Mccree, C. , & Angeli, P. (2013). Inter‐agency joint protocols for safeguarding children in social care and adult mental‐health agencies: A cross‐sectional survey of practitioner experiences. Child & Family Social Work, 18(2), 149–158. 10.1111/j.1365-2206.2011.00816.x

[hsc12978-bib-0060] Welland, C. , & Ribner, N. (2010). Culturally specific treatment for partner‐abusive Latino men: A qualitative study to identify and implement program components. Violence and Victims, 25(6), 799–813. 21287968 10.1891/0886-6708.25.6.799

[hsc12978-bib-0061] Willis, D. , Hawkins, J. W. , Pearce, C. W. , Phalen, J. , Keet, M. , & Singer, C. (2010). Children who witness violence: What services do they need to heal? Issues in Mental Health Nursing, 31(9), 552–560. 10.3109/01612841003721461 20701417

[hsc12978-bib-0062] Zlotnick, C. , Capezza, N. M. , & Parker, D. (2011). An interpersonally based intervention for low income pregnant women with intimate partner violence: A pilot study. Archives of Women's Mental Health, 14(1), 55–65. 10.1007/s00737-010-0195-x PMC3042850 21153559

